# Correlation of Model for End Stage Liver Disease (MELD), MELD-Sodium (MELD-Na), and Child-Turcotte-Pugh (CTP) Score With Frailty in Patients With Hepatitis C Virus (HCV) Related Cirrhosis

**DOI:** 10.7759/cureus.40574

**Published:** 2023-06-17

**Authors:** Muhammad Qaiser Panezai, Raja Taha Yaseen, Ghulamullah Lail, Muhammad Ali Khalid, Hina Ismail, Zain Majid, Danish Kumar, Saleem Shahzad, Syed Mudassir Laeeq, Nasir Hassan Luck

**Affiliations:** 1 Hepatogastroenterology, Sindh Institute of Urology and Transplantation, Karachi, PAK

**Keywords:** meld-sodium, model for end stage liver disease (meld), frailty, correlation, hepatitis c

## Abstract

Introduction: The model for end stage liver disease (MELD), model for end stage liver disease-sodium (MELD Na), and Child-Turcotte-Pugh (CTP) score are independent predictors of mortality in cirrhotic patients. Approximately 43% of cirrhotic patients with advanced disease are frail and can have detrimental effects on the disease prognosis and survival including delisting from the transplant list and increased risk of post-transplant complications. Therefore, our aim was to determine the correlation of MELD, MELD-Na, and CTP score with frailty in patients with hepatitis C virus (HCV) related cirrhosis.

Methods: This cross-sectional study was conducted at the Department of Hepato-gastroenterology, Sindh Institute of Urology and Transplantation from 1^st^ January 2022 to 30^th^ June 2022. All the patients of either gender aged between 18 and 70 years with serological evidence of HCV and features of cirrhosis on ultrasound abdomen were included in the study. Patients with conditions over estimating frailty were excluded from the study. Liver Frailty Index (LFI) was calculated using grip strength measured in kilograms, timed chair stands, and balance testing. CTP and MELD-Na scores for each patient were also recorded. All the data were analyzed using SPSS version 22.0 (IBM Corp., Armonk, NY). The correlation of MELD, MELD-Na, and CTP with LFI was analyzed using the Pearson correlation coefficient and a p-value < 0.05 was considered statistically significant.

Results: A total of 274 patients were included in the study. Out of them, 185 (67.5%) were males. The mean CTP score was 8.1 + 2.1, MELD score of 13.6 + 7.1, MELD-Na score of 15 + 6.6, and LFI of 4.1 + 0.83. LFI was found to be weakly correlated with MELD (r = 0.278) (p < 0.001), MELD-Na score (r = 0.41) (p < 0.001), and CTP score (r = 0.325) (p < 0.001).

Conclusion: Weak correlation was noted between LFI, CTP, MELD, and MELD-Na scores in HCV-associated chronic liver disease. Therefore, frailty along with MELD, MELD-Na, and CTP must be assessed before considering the patients for liver transplantation.

## Introduction

Cirrhosis can occur as a result of sustained liver injury that can be multifactorial with etiologies ranging from viral hepatitis to autoimmune hepatitis, alcoholic hepatitis, and drug-related liver injury [1]. One of the leading causes of cirrhosis is chronic hepatitis C virus (HCV). Despite advancements in health and medical sciences, approximately 71 million of the world's population is infected with HCV [2]. Cirrhosis is histologically characterized by regenerative nodules and fibrous bands found on the examination of liver tissue [3]. Numerous complications may develop as a consequence of cirrhosis including portal hypertension, ascites, variceal hemorrhages, and hepatocellular carcinoma [4].

 Frailty is defined as a limited physiological reserve, reduced resistance to stressors, and increased vulnerability to adverse outcomes [5]. Frailty affects around 40% of cirrhotic patients. It is associated with psychosocial effects, increased rates of hepatic decompensation, hospital stay, delisting from the transplant list, and post-transplant ramifications [6-7]. Additionally, frailty similar to the model for end-stage liver disease (MELD) and MELD-sodium (MELD-Na) has also gained attention as a predictor of mortality in cirrhosis. However, the latter two do not estimate the functional reserve of cirrhotic patients [8-9].

 Up till now, different methods have been predicted and utilized for the assessment of frailty including the Liver Frailty Index (LFI), Fried Frailty Criteria, Short Performance Battery, and Clinical Frailty Index. All these scales are comparable in predicting frailty in cirrhosis [10]. However, LFI is a purely performance-based bedside index and its components (hand grip strength, chair stands, and balance) are measured objectively [11].

 As MELD, MELD-Na, Child-Turcotte-Pugh (CTP) score, and Frailty, are predictors of mortality; we, therefore, aim to evaluate the correlation between these prognostic models. As MELD, MELD-Na, and CTP scores are based on invasive and costly laboratory parameters that predict only the hepatic consequences in cirrhotic patients, LFI has emerged as an office-based objective tool that can be utilized for the prediction of extrahepatic consequences in cirrhotic patients. Therefore, this study not only helped us to determine the utility of LFI as an adjunct to the already available prognostic scores but also facilitated us in predicting a correlation between these scores.

Therefore, the main objective of our study was to determine the correlation of MELD, MELD-Na, and CTP with frailty in patients with HCV-related cirrhosis.

## Materials and methods

Sample size calculation

Assuming the correlation coefficient between MELD-Na and LFI (r=0.3) was calculated in a pilot study with a power of 80% and a 5% confidence level, a total of 85 patients were required for the study.

Methodology

This cross-sectional study was conducted at the Department of Hepato-gastroenterology, Sindh Institute of Urology and Transplantation from 1st January 2022 to 30th June 2022. All patients aged between 18 and 70 years of either gender with serological evidence of HCV (i.e. anti-HCV reactive) and features of cirrhosis on ultrasound abdomen were enrolled in the study. While, the patients suffering from conditions that overestimate frailty, e.g. cardiopulmonary disease, osteoarthritis, etc., or those with altered mentation or a history of hepatocellular carcinoma were excluded from the study.

Data collection procedure

After the approval from the Ethical Review Board (ERC), Sindh Institute of Urology and Transplantation (SIUT), informed consent was taken from all the patients before enrolment in the study. All the HCV-related cirrhotic patients as per inclusion criteria presenting to outpatient or admitted to the Department of Hepatogastroenterology, Sindh Institute of Urology and Transplantation, Karachi were included in the study. LFI was calculated by the researcher for grip strength measured in kilograms, timed chair stands (height of chair will be 42 cm from ground surface) in seconds, and balance testing measured in seconds as per operational definition. Laboratory work up including total bilirubin, creatinine, sodium, and international normalized ratio (INR) was sent to calculate CTP, MELD, and MELD-Na during the same visit as per the operational definition. All the findings were recorded in the predesigned Proforma.

 Statistical analysis

All the data were entered and analyzed using Statistical Package for the Social Sciences (SPSS) version 22.0 (IBM Corp., Armonk, NY). Mean and standard deviation were computed for the continuous variables including age, MELD, MELD-Na, LFI, CTP score, and other lab parameters; while frequency and percentages were calculated for the categorical variables including gender. Categorical variables were analyzed using the chi-square test while continuous variables were analyzed using the student’s t-test. The correlation of MELD, MELD-Na, and CTP scores with LFI was analyzed using the Pearson correlation coefficient. A p-value ≤ 0.05 was considered statistically significant.

## Results

A total of 274 patients were included in the study. Out of them, 185 (67.5%) were males. One hundred and thirty (47.4%) patients belonged to CTP class B. Forty-two (15.3%) patients had previous history of portosystemic encephalopathy. Esophageal varices were observed in 156 (57%) patients while 21 (7.7%) patients had history of spontaneous bacterial peritonitis. Mean CTP score was of 8.1 + 2.1, MELD score of 13.6 + 7.1, MELD-Na score of 15 + 6.6, and LFI of 4.1 + 0.83 (Table 1).

**Table 1:** Operational definition of cirrhosis of liver, LFI, MELD, and MELD-Na. MELD, model for end stage liver disease; MELD-Na, model for end stage liver disease-sodium; LFI, liver frailty index

Operational definitions
Cirrhosis of liver [12]: The patient with liver cirrhosis will be identified on abdominal ultrasound findings. The presence of three or more of the following findings will be considered as the presence of liver cirrhosis. 1. Altered echo texture of liver. 2. Irregular margins. 3. Spleen size more than 12 cm. 4. Portal vein diameter more than 12 mm. 5. Presence of free fluid in the abdomen on ultrasound.
Liver Frailty Index [LFI] [8]: Liver frailty will be calculated using LFI [8] which compromises grip strength: the average of three trials in the subject’s non-dominant hand using a hand dynamometer will be measured in kilograms. A single, hand dynamometer will be used to record the grip strength of each patient. Timed chair stands: will be measured as the number of seconds it takes to do five chair stands with the subject’s arms folded across the chest. A single chair having a height of 42 cm from the ground surface will be used. Balance testing: will be measured as the number of seconds that the patient can balance in the following three positions: feet placed side-to-side, semi tandem, and tandem. (Note: maximum time period for balance testing in each position will be 10 s). LFI [8] will be measured by putting the following values into the equation: LFI [8] = (-0.330 × gender-adjusted grip strength) + (-2.529 × number of chair stands per second) + (-0.040 × balance time) + 6
Model for end stage liver disease (MELD) [13]: It is a scoring system that predicts short-term mortality in cirrhotic patients and is calculated using the clinical parameters, i.e. creatinine, bilirubin, and international normalized ratio (INR). MELD [13]= 0.957 × ln (Cr) + 0.378 × ln (bilirubin) + 1.120 × ln (INR) + 0.643 (score range: 6-40)
Model for end stage liver disease-sodium (MELD-Na) [13]: MELD-Na is similar to MELD but it includes serum sodium. MELD-Na [13] = MELD score - Na - 0.025 x MELD x (140-Na) + 140

**Figure 1:**
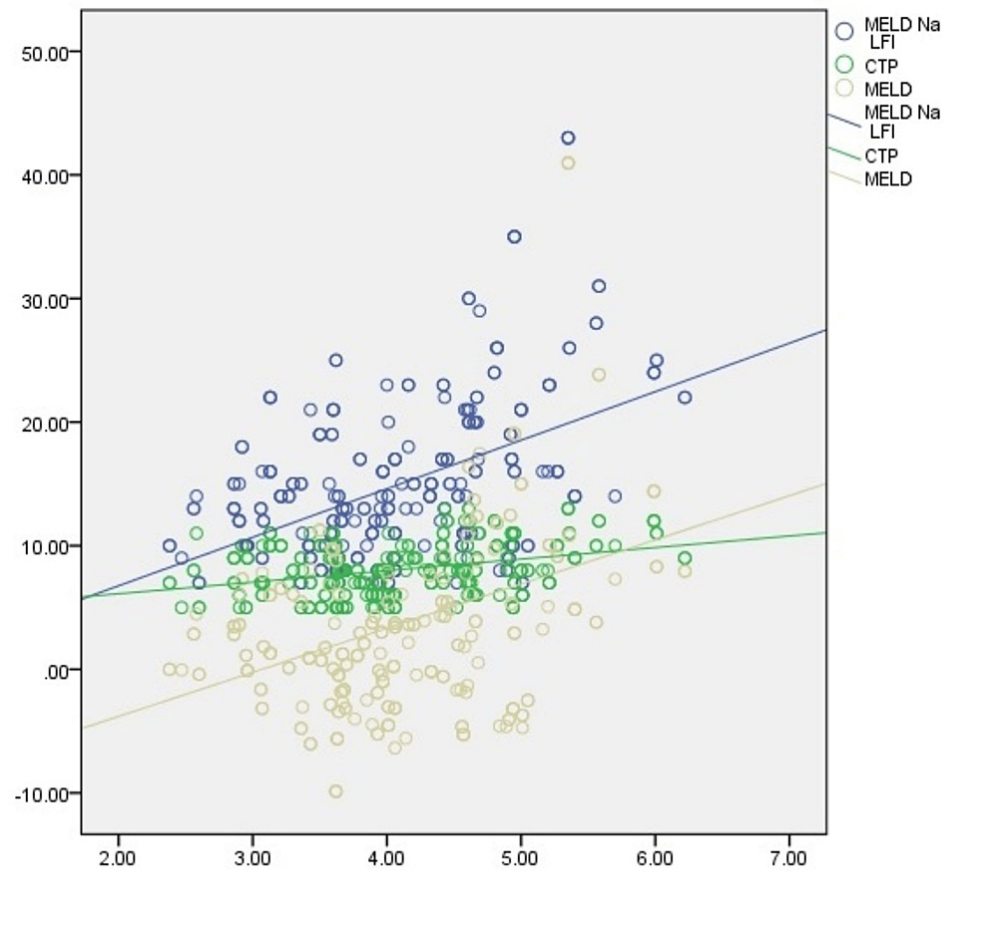
Pearson correlation plot showing correlation of frailty with CTP (r=0.327), MELD (r=0.278), and MELD-Na (r=0.410) in HCV-related chronic liver disease. CTP, Child-Turcotte-Pugh; MELD, model for end stage liver disease; MELD-Na, model for end stage liver disease-sodium; LFI, liver frailty index; HCV, hepatitis C virus

On stratification, increased frailty was observed in females, in patients with advanced age, and in patients with raised TLC, serum creatinine, serum bilirubin, INR, CTP, MELD, and MELD-Na. Frailty was also found to be significantly increased in patients with low sodium and serum albumin (Table 2). LFI was found to be weakly correlated with MELD score (r = 0.278) (p < 0.001), MELD-Na score (r = 0.41) (p < 0.001), and CTP score (r = 0.325) ( p < 0.001) (Table 3) (Figure 1).

**Table 2:** Baseline characteristics of the studied population (n=274). CTP, Child-Turcotte-Pugh; MELD, model for end stage liver disease; MELD-Na, model for end stage liver disease-sodium; LFI, liver frailty index; SD, standard deviation; INR, international normalized ratio; BMI, body mass index

Study population (n=274)			n (%)
Mean age (years ± SD)			46 + 13
Gender		Male	185 (67.5)
		Female	89 (32.5)
History of ascites		Present	168 (61.3)
		Absent	106 (38.7)
History of hepatic encephalopathy		Present	42 (15.3)
		Absent	232 (84.7)
Esophageal varices		Present	156 (56.9)
		Absent	118 (43.1)
History of spontaneous bacterial peritonitis		Present	21 (7.7)
		Absent	253 (92.3)
Hemoglobin (g/dL)			10.1 ± 2.15
Total leucocyte count (x10^9^/L)			5.5 ± 2.8
Platelet count (x10^9^/L)			97.8 ± 62.8
Serum creatinine (mg/dL)			1.0 ± 0.89
Total bilirubin (mg/dL)			2.9 ± 4.3
Albumin (g/dL)			2.9 ± 0.7
INR			1.3 + 0.32
Serum sodium (mEq/L)			137 + 5.5
MELD score			13.6 + 7.1
MELD-Na score			15 + 6.6
CTP class	A		68 (24.8)
	B		130 (47.4)
	C		76 (27.2)
CTP score			8.1 + 2.1
Handgrip (kg)			20.4 + 14
Chair stands (s)			14.8 + 10.3
Balance (s)			9.8 + 0.8
BMI			21.5 + 8.6
LFI			4.1 + 0.8

**Table 3:** Comparison of baseline variables in terms of liver frailty (n=274). CTP, Child-Turcotte-Pugh; MELD, model for end stage liver disease; MELD-Na, model for end stage liver disease-Na; LFI, liver frailty index

Variable		LFI (>4.5) Mean ± SD	LFI (< 4.5) Mean ± SD	p-value
Age		52.1 ± 10.6	43 ± 13	≤0.001
Gender	Males	46(51)	139 (75.5)	≤0.001
	Females	44 (49)	45 (24.5)	
Hemoglobin (g/dL)		9.2 ± 1.7	10.5 ± 2.2	≤0.001
Total leucocyte count (x10^9^/L)		6.8 ± 3.3	4.8 ± 2.3	≤0.001
Platelet count (x10^9^/L)		102 ± 48.6	95 ± 68	0.46
Total bilirubin (mg/dL)		4.8 ± 7.0	2.0 ± 1.4	≤0.001
Serum albumin (g/dL)		2.7 ± 0.6	3.1 ± 0.7	≤0.001
Serum creatinine (mg/dL)		1.5 ± 1.3	0.8 ± 0.3	≤0.001
Serum sodium (mEq/L)		134 ± 7.6	138 ± 3.6	≤0.001
International normalized ratio (INR)		1.4 ± 0.4	1.3 ± 0.2	≤0.001
CTP score		9.0 ± 2.2	7.6 ± 1.9	≤0.001
MELD score		7.8 ± 9.5	1.7 ± 4.5	≤0.001
MELD-Na score		19.4 ± 8.3	12.8 ± 4.3	≤0.001

## Discussion

 It is a known fact that cirrhosis not only impairs liver function but also imposes a huge impact on the extrahepatic functions of the body [14]. Extrahepatic dysfunction is commonly a neglected subject in patients with cirrhosis. One way of assessing the extrahepatic effects of cirrhosis is to assess the general well-being of a patient. Various methods are available for the assessment of the extrahepatic impact of cirrhosis including the Eastern Cooperative Oncology Group (ECOG) status and quality of life questionnaires, sarcopenia, etc. Instead, all these methods are subjective. Frailty as assessed by the LFI is an objective index that can be utilized for the estimation of extrahepatic consequences of cirrhosis [8, 15]. It has also been observed that frailty is not an uncommon feature in cirrhosis [16-19].

 Patients having the same grade of hepatic dysfunction might have different degrees of frailty. For instance, if we compare two cirrhotic patients having the same MELD and MELD-Na, one patient is able to carry out his daily activities and has no history of decompensation, while the other one can barely move without the support and has massive ascites. No doubt, both have equal MELD-Na but it is easy to predict that the latter patient has more chances of further decompensation, hospitalization, and early death. Similarly, previous studies not only demonstrated the association of frailty with increased risk of cirrhosis progression and death but also predicted the role of a combination of both frailty and MELD-Na as a better prognosticator of mortality in patients with cirrhosis [8, 16].

 It has been established that CTP, MELD, and MELD-Na encompass the hepatic manifestation of cirrhosis and can be utilized to predict mortality in cirrhotic patients [20-21]. Similarly, LFI has also promised its role in predicting mortality in cirrhotic populations [7-9, 16]. As all these parameters are predictors of mortality in cirrhosis, it was important to explore the correlation between both the hepatic and extrahepatic effects of cirrhosis.

 In our population, about 39% of cirrhotic patients were frail. On further analysis, frailty was found to have a weak correlation with MELD, MELD-Na (r = 0.41), and CTP (r = 0.327). First, as shown by the results of this study, it has been verified that frailty is common in cirrhotics. Second, the conventional scores used for the assessment of liver disease are unable to portray the extrahepatic effects of cirrhosis. Third, both hepatic and extrahepatic components need to be addressed differently. Certainly, liver-related complications cannot be reversed without liver transplantation, but frailty can be reversed by improving nutrition and exercise [22-23]. Therefore, this neglected aspect of cirrhosis can have an enormous impact on the survival of the patient with cirrhosis, if treated accurately [23].

Up till now, the criteria for the induction of liver transplantation are solely based on the advanced MELD and MELD-Na score. But, as this study has shown only the weak correlation of frailty with MELD, MELD-Na, and CTP; therefore, frailty must not only be assessed in all the patients with cirrhosis but frail patients must also be considered for liver transplantation even if their hepatic scores are borderline for liver transplantation (Table 4).

**Table 4:** Correlation of frailty with CTP, MELD, and MELD-Na in HCV-related chronic liver disease. CTP, Child-Turcotte-Pugh; MELD, model for end stage liver disease; MELD-Na, model for end stage liver disease-sodium; LFI, liver frailty index; HCV, hepatitis C virus

Variable		CTP score	MELD	MELD-Na
LFI	Pearson correlation (r)	0.327	0.278	0.410
	p-value	<0.001	<0.001	<0.001

 We acknowledge certain shortcomings of our study. At first, frailty, MELD, and MELD-Na were evaluated only once and were not followed with the passage of time. Second, we did not estimate and correlate the level of sarcopenia in these patients as it may impact frailty. Third, we did not segregate patients with compensated and decompensated cirrhosis.

## Conclusions

Keeping in view the weak correlation of hepatic and extrahepatic components of cirrhosis, frailty along with MELD-Na and CTP must be assessed before considering patients for liver transplantation. Numerous valuable lives may be lost if the hepatic component is considered and the extrahepatic component remains neglected. Multicentric studies with larger sample sizes are required not only to determine the role of frailty in predicting the extrahepatic consequences of cirrhosis but also to predict the non-invasive markers suggestive of frailty in these patients.
